# Bats on a Budget: Torpor-Assisted Migration Saves Time and Energy

**DOI:** 10.1371/journal.pone.0115724

**Published:** 2014-12-31

**Authors:** Liam P. McGuire, Kristin A. Jonasson, Christopher G. Guglielmo

**Affiliations:** Department of Biology, Advanced Facility for Avian Research, Western University, London, Ontario, Canada; Pennsylvania State University, United States of America

## Abstract

Bats and birds must balance time and energy budgets during migration. Migrating bats face similar physiological challenges to birds, but nocturnality creates special challenges for bats, such as a conflict between travelling and refueling, which many birds avoid by feeding in daylight and flying at night. As endothermic animals, bats and birds alike must expend substantial amounts of energy to maintain high body temperatures. For migratory birds refueling at stopovers, remaining euthermic during inactive periods reduces the net refuelling rate, thereby prolonging stopover duration and delaying subsequent movement. We hypothesized that bats could mitigate similar ambient-temperature dependent costs by using a torpor-assisted migration strategy. We studied silver-haired bats *Lasionycteris noctivagans* during autumn migration using a combination of respirometry and temperature-sensitive radiotelemetry to estimate energy costs incurred under ambient temperature conditions, and the energy that bats saved by using torpor during daytime roosting periods. All bats, regardless of sex, age, or body condition used torpor at stopover and saved up to 91% of the energy they would have expended to remain euthermic. Furthermore, bats modulated use of torpor depending on ambient temperature. By adjusting the time spent torpid, bats achieved a rate of energy expenditure independent of the ambient temperature encountered at stopover. By lowering body temperature during inactive periods, fuel stores are spared, reducing the need for refuelling. Optimal migration models consider trade-offs between time and energy. Heterothermy provides a physiological strategy that allows bats to conserve energy without paying a time penalty as they migrate. Although uncommon, some avian lineages are known to use heterothermy, and current theoretical models of migration may not be appropriate for these groups. We propose that thermoregulatory strategies should be an important consideration of future migration studies of both bats and birds.

## Introduction

Migrating bats, and the great majority of migrating birds, make their flights at night when conditions appear to be most favourable for flying long distances [1]. Bats and birds typically land at stopover sites before sunrise, and birds (being diurnal except for species like owls or nightjars) forage to refuel for the next flight. Bats, being generally nocturnal, do not feed during the day and roost until night [2] (although diurnal activity in migrating noctules *Nyctalus noctula* is a notable exception [3]). Lost opportunity to refuel during the day places an added time constraint on migrating bats that could impede progress of their journey because they must use the nocturnal active period either to travel or refuel [2]. However, the well-described physiological capacity of bats to enter torpor (reduced body temperature and metabolic rate) during the day could confer an energy advantage that would allow them to migrate efficiently, and perhaps more quickly than most birds.

For birds, thermoregulatory costs during inactive periods at stopover can substantially influence the energetics and timing of migration [4]. Birds typically require several days (or even weeks) at a stopover to rebuild their fuel stores [5]. Theoretical and empirical evidence indicates that birds spend more time at stopover sites than in flight, and that stopover periods account for approximately two-thirds of the total energy expenditure during migration [4], [6]. Energy costs during stopover are strongly linked to ambient temperature (T_a_) because the cost of maintaining euthermic body temperature increases as T_a_ decreases below the thermal neutral zone [4]. Thermoregulatory costs decrease net refuelling rate and prolong stopover because some nutrients accumulated at stopover are used to meet immediate energy demands rather than being conserved for future flights.

Torpor use by migrating bats was first described in spring-migrating silver-haired bats *Lasionycteris noctivagans*, particularly in response to inclement weather (<8°C or raining) [7]. Similarly, migrating male hoary bats *Lasiurus cinereus* readily used torpor when cold challenged [8]. Recently, we used automated radiotelemetry to study stopover behaviour of migrating bats, and observed that it was quite different from songbirds refueling at the same site [9], [10]. While birds frequently remained for several days, most bats arrived at dawn and departed at dusk the same day when weather conditions permitted (i.e., not raining). Extended foraging bouts were uncommon in bats, contrary to typical hyperphagic refuelling observed in migrating birds [11]. We hypothesized that bats used torpor to minimize non-flight costs of migration, reducing the need for foraging and sparing nutrient stores to fuel subsequent migratory flight. We proposed the term ‘torpor-assisted migration’ to describe this strategy [10] whereby bats use torpor in anticipation of future energy expenditures and not simply in response to immediate energy emergency.

The objective of our study was to test the torpor-assisted migration hypothesis by describing thermoregulatory patterns of migrating bats. Further, we developed the implications of heterothermy for the energetics of migration, especially in context of comparisons with homeothermic migrants and the implications for overall migration patterns.

## Materials and Methods

**Figure 1:**
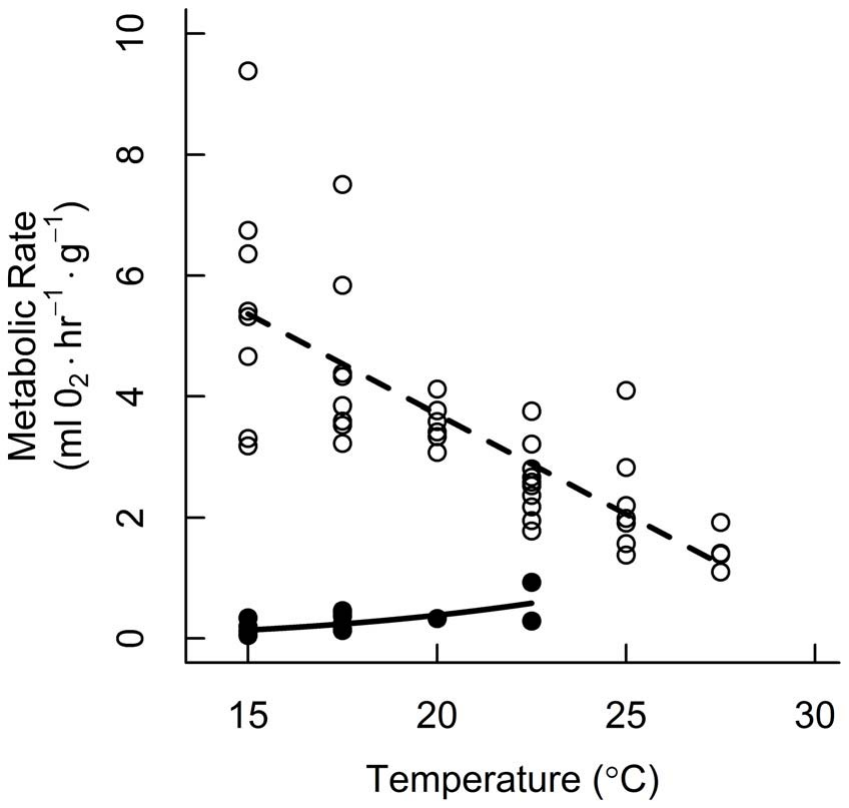
Torpid and euthermic metabolic rates measured by open flow respirometry. Silver-haired bats were held at a range of temperatures similar to local conditions. Open circles and dashed regression line indicate resting metabolic rate (RMR) and filled circles and solid regression line indicate torpid metabolic rate (TMR).

We used mist nets to capture migrating silver-haired bats during autumn migration (August 18 – Sept 15, 2011) at the Long Point Bird Observatory, Long Point, ON, Canada (42.6°N 80.4°W). See [10] for descriptions of the study species and study site. There were two experimental groups in our study. In group one, silver-haired bats were held in respirometry chambers for the daytime roosting period to measure euthermic resting metabolic rate (RMR) and torpid metabolic rate (TMR) across a range of temperatures. In group two, we attached temperature-sensitive radio-transmitters to bats to record skin temperature (T_sk_) in free-living individuals. Bats used for respirometry measurements (group one) were not used for radio-tracking (group two). We used relationships between T_a_ and either TMR or RMR from respirometry (group one; Fig. 1) to predict roosting field energy expenditure of free-living bats (group two). All calculations and statistical analyses were conducted with R statistical software [12]. All research activities were approved by the University of Western Ontario Council on Animal Care (protocol 2010-020) and the Ontario Ministry of Natural Resources (licence no. 1063353).

### Respirometry

We used flow-through respirometry to measure RMR and TMR of silver-haired bats at temperatures from 15.0–27.5°C. The range of temperatures was selected to approximate T_a_ at the study site. Bats were held at one temperature in the morning, before increasing or decreasing T_a_ by 2.5°C for a second metabolic trial in the afternoon. Other than the 2.5°C temperature change, bats were not disturbed during respirometry trials. Bats were exposed to each experimental temperature for at least 5 hours. Upon removal from respirometry chambers, we used a two channel thermocouple (±1°C; Model 4026 Type-K Traceable Expanded Range Thermometer, Control Company, Friendswood, TX) to simultaneously measure skin and rectal temperature.

Respirometry trials took place during the inactive period, beginning shortly after sunrise and continuing until dusk. Metabolic chambers were constructed out of 1.12 L metal canisters with a hanging piece of plastic mesh for bats to roost. Chambers were housed in a dark, temperature-controlled cabinet (+/−0.2°C; model PTC-1 with PELT-5 temperature controller; Sable Systems, Las Vegas, NV, USA). Incurrent ambient air was pumped through a column of Drierite to remove water vapour, before passing through needle valves (air flow manifold MF-8; Sable Systems), which supplied chambers with a constant flow of ∼200 mL·min^−1^. Flow rate was measured with a mass flow meter (840L; Sierra Instruments, Monterey, CA, USA) after passing through metabolic chambers and a second Drierite column to remove any water vapour produced by the study subjects. Excurrent air was subsampled at 90 mL·min^−1^ (Gas analyzer sub-sampler v2.0 Sable Systems) before gas concentrations were measured with CO_2_ (model CA-2A; Sable Systems) and O_2_ analyzers (model FC-1B; Sable Systems). We used a multiplexer to monitor each chamber for 10 min before switching to the next chamber. Up to four metabolic chambers were used simultaneously. A 10 min baseline of reference airflow was measured after each round of recordings. All data were recorded using an analog-to-digital converter (UI-2; Sable Systems) connected to a laptop computer. Fractional concentrations of O_2_ and CO_2_ were lag- and drift-corrected and VO_2_ (ml·min^−1^), and VCO_2_ (ml·min^−1^) were calculated using equations 11.7 and 11.8 from [13] using ExpeData analytical software (v1.1.15, Sable Systems).

We searched for the lowest average 200 s period of VO_2_ in each 10 min recording interval. At T_a_ ≤22.5°C periods of TMR and RMR were visually distinguishable. At higher temperatures we were not able to identify periods of torpor. For each individual bat at each experimental temperature, we averaged intervals with the two lowest mean VO_2_ measurements for both TMR and RMR, as applicable. RMR and TMR measurements were used to develop predictive equations for estimating metabolic rate of free-living bats in group 2 based on T_a_.

We used linear mixed effects models with individual bats treated as random factors to account for repeated measures. Our model selection process followed the methods described by [14]. We fit an initial model with temperature, sex, age, and temperature order (whether temperature was increased or decreased in the afternoon), and all 2- and 3-way interactions of temperature, sex, and age. We removed one parameter at a time, comparing nested models with likelihood ratio tests. We sequentially removed the least significant term until only significant terms remained. For TMR we log transformed both metabolic rate and T_a_ values to account for the curvilinear response of TMR with T_a_.

### Telemetry

In group two, T_sk_ in free-living bats was quantified with temperature-sensitive radio-transmitters (±0.1°C, 0.38 g, <4% body mass; Pip3, Lotek Wireless, Newmarket, Ontario, Canada). This data was later used to identify periods of torpor (see Energy Calculations below). Transmitters were calibrated by the manufacturer at nine points (≤5°C intervals) from −1.8 to 37.0°C. Upon capture, we weighed each bat (±0.1 g) and used a quantitative magnetic resonance (QMR) body composition analyzer (EchoMRI-B; Echo Medical Systems, Houston, TX, USA) to determine fat mass and lean body mass (±0.01 g) [10], [15]. We trimmed fur in the interscapular region close to the skin and affixed transmitters with ostomy bonding cement (Torbot; Cranston, RI, USA) and then released the bat. Shortly after dawn we tracked bats to their day roosts using 3-element Yagi antennas and recorded T_sk_ every ∼30 seconds with a datalogging receiver (SRX400; Lotek Wireless). The placement of the receiver and antenna ensured continuous recording until the bat departed from the roost. Ambient temperature was recorded at a nearby (6 km) weather station operated by Bird Studies Canada (Port Rowan, ON).

We used QMR body composition data to test for the effect of body condition on torpor expression (see Energy Calculations section below for method of identifying periods of torpor). We used general linear models to test for effects of fat mass, controlling for body mass and mean daytime T_a_ on the number of torpor bouts, mean torpor bout duration, maximum torpor bout duration, time spent euthermic, or time spent below normal body temperature (not euthermic). For each dependent variable we began with a model including all main effects, two- and three-way interactions. We sequentially removed the least significant term and re-evaluated the model until only significant (p<0.05) terms remained.

### Energy Calculations

We estimated energy expenditure by applying regression equations of metabolic rate (RMR or TMR) and T_a_ from bats in group one to periods of torpor and euthermia observed in free-living bats (group two), accounting for warming and cooling costs. Energy expenditure was calculated only for daytime periods. For the purposes of our calculations, a day began either at sunrise, or the moment a bat was first located, and continued until either sunset or when the bat returned to euthermia if the bat had not aroused at sunset. We classified each T_sk_ measurement as either euthermic, warming, cooling, or torpid. For each bat we identified a period (>50 min except for one bat where only 20 min was possible) of stable euthermic body temperature and calculated the mean T_sk_ (range 30.3–36.6°C). We defined the torpor cut-off by taking the lower limit of the 99% confidence interval for euthermic body temperature and subtracting 3°C, as described by [16]. This method allows for individual variation in T_sk_ due to transmitter application differences (e.g. thickness of remaining fur and/or adhesive). Warming was identified at transitions from torpor to euthermia, where the rate of temperature increase was greater than 9°C hr^−1^, the minimum warming rate for bats reported by [17]. Cooling is a passive process (but see [18] for variation in metabolic rate during cooling) and therefore the rate is dependent on ambient conditions. Consequently we did not define a cut-off cooling rate, but rather defined cooling as the upper 10^th^ percentile of the distribution of (T_sk_ - T_a_) during non-euthermic periods (excluding warming), essentially assuming bats are torpid most of the time and cooling only briefly. Torpor bouts <15 min in duration were excluded and conservatively classified as euthermic. Warming costs were calculated as per [19] and [8], assuming T_sk_ measured by radiotransmitters is similar to body temperature [20, our data showing no difference in T_sk_ and rectal temperature], and calculating thermal conductance from allometric scaling [21]. Cooling costs were calculated as 67.2% of warming costs [22], excluding heat loss to the environment [8]. When necessary, we used the oxyjoule equivalent [13] to convert from units of mL O_2_ h^-1^ to joules, and subsequently to Watts.

Infrequently, T_a_ extended beyond the range of temperatures included in the TMR or RMR predictive equations. For RMR, if T_a_>27.5°C (maximum observed RMR T_a_ = 29.9°C) we used the predicted value for 27.5°C. If RMR T_a_<15°C (minimum observed RMR T_a_ = 10.8°C) we extrapolated the predictive equation. For TMR, if T_a_ was outside the range of the predictive equations (minimum observed TMR T_a_ = 5.9°C, maximum observed TMR T_a_ = 25.7°C) we extrapolated the predictive equation.

## Results

We caught 24 silver-haired bats (10.6±0.2 g) for use in respirometry (S1 Table). We obtained 43 values of RMR from 22 bats, and 15 values of TMR from 9 bats (Fig. 1). Metabolic rate was not affected by sex, age, the direction of T_a_ change, or any interactions (all p>0.05). Ambient temperature was the only significant predictor of mass-specific metabolic rate (RMR: *F*_1,20_ = 57.43, *P*<0.001; TMR: *F*_1,5_ = 9.14, *P* = 0.029). The predicted relationship for euthermic bats was: Metabolic rate (ml O_2_·hr^−1^·g^−1^)  = 10.326–0.331*T_a_(°C). The predicted relationship for torpid bats was: ln(Metabolic rate [ml O_2_·hr^−1^·g^−1^])  = −11.559+3.539*ln(T_a_[°C]). Skin and rectal temperatures (including torpid and euthermic bats, range 16–37°C) were not different as measured by the two-channel thermocouple (paired t-test: *t*_24_ = 0.22, *P* = 0.82).

**Figure 2:**
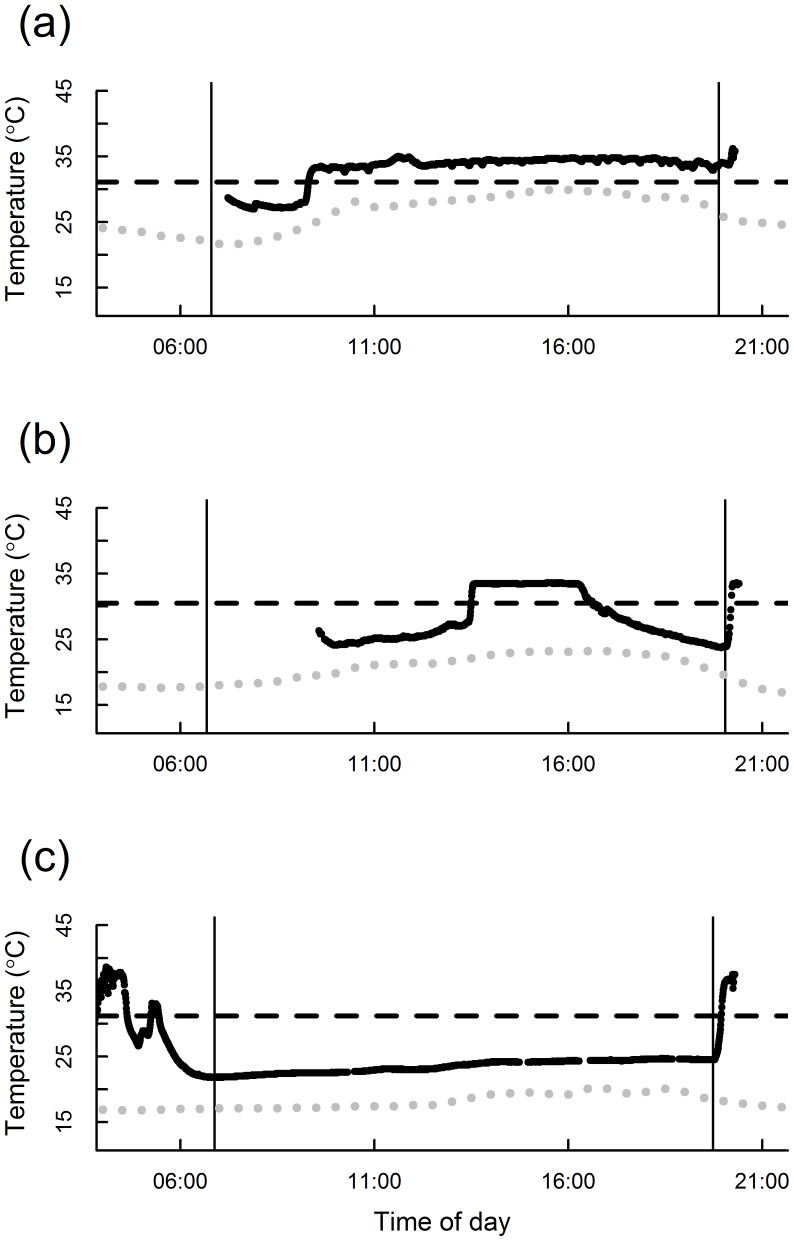
Temperature profiles of free-living silver-haired bats during autumn migration. Vertical lines indicate sunrise and sunset, horizontal dashed line indicates the individually determined threshold below which the bat was considered torpid (see Materials and Methods), black dots indicate skin temperature, and gray dots indicate ambient temperature. The amount of time spent torpid depended on ambient conditions. Mean ambient temperature over the period of observation was 27.4°C (Sept 3), 21.9°C (Aug 28), and 18.3°C (Sept 8) in panels (a), (b), and (c) respectively. Accordingly, in (a) the bat spent only a brief period of the morning in torpor, whereas at cooler temperatures bats spent most (b) or all (c) of the day torpid.

We attached radiotransmitters to 25 silver-haired bats (10.7±0.2 g), all of which used torpor regardless of sex or age. We obtained sufficient data records for detailed analysis of 16 silver-haired bats: seven sub-adult male, six sub-adult female, two adult male, one adult female (S2 Table). Two bats were present on consecutive days, resulting in 18 bat-days for analysis. Duration of torpor varied widely (Fig. 2). Bats entered 1–3 torpor bouts per day, although the most common pattern was a morning and evening bout (Fig. 2b). On cooler days, some bats remained torpid for the entire day, arousing at sunset to depart from the site (Fig. 2c). Among torpid bats, minimum T_sk_ was 2.6±0.4°C above T_a_.

We did not observe any torpor bouts in free-living bats when T_a_ was>25.7°C, similar to the intersection point of the TMR and RMR regression equations (27.6°C). QMR body composition data was available for 14 of the bats with sufficient telemetry records for analysis. Fat mass (0.99±0.08 g; 9.24±0.61% of body mass) was not related to any measures of torpor expression (number of torpor bouts, mean bout duration, maximum bout duration, time spent below normal body temperature; *P*>0.05 in all cases).

**Figure 3:**
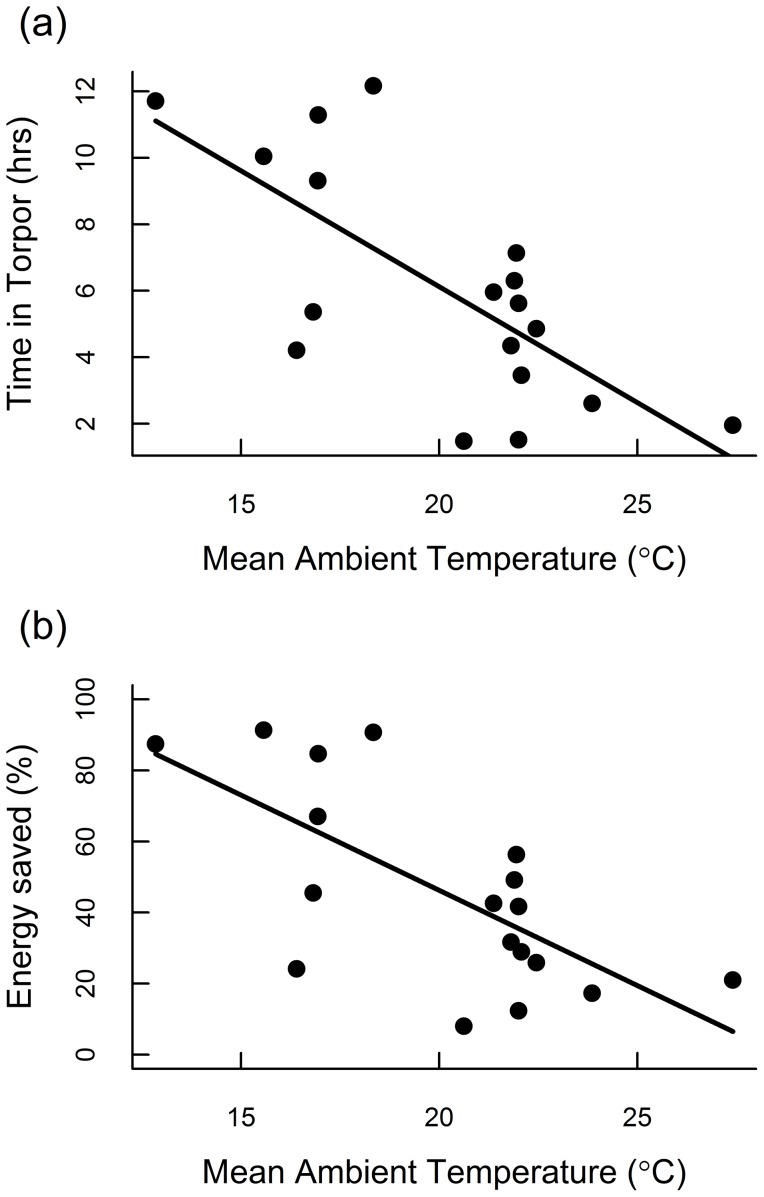
Relative energy savings of daytime torpor use during stopover. (a) On cooler days, when the energetic cost of defending normal body temperature would be greater, bats spent more time in torpor. (b) Bats saved 12–91% of the estimated energy required to remain euthermic, and saved more energy on cooler days.

**Figure 4:**
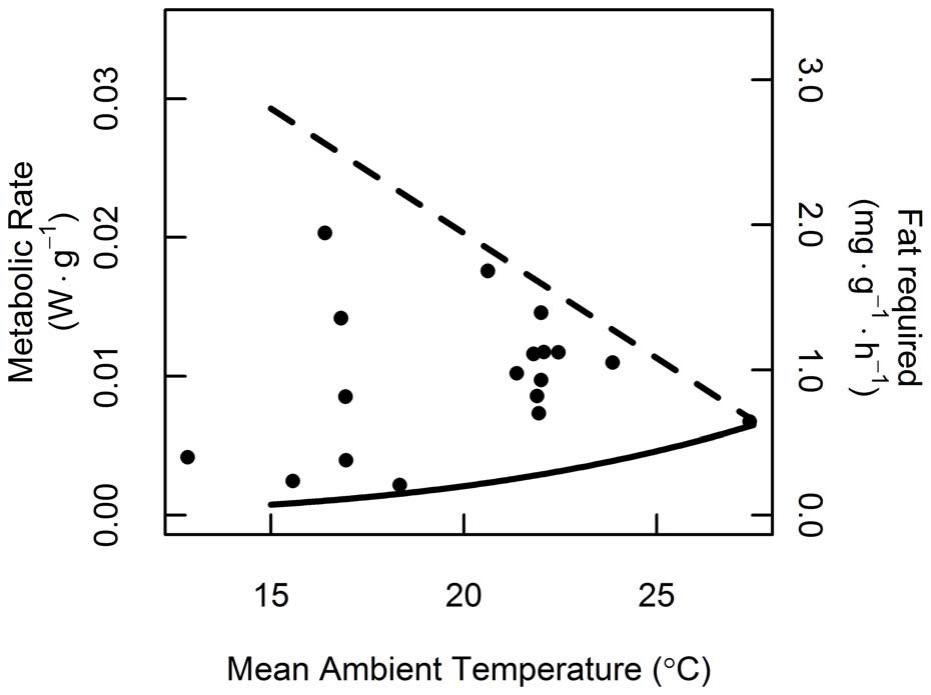
Estimated field metabolic rate of migrating bats calculated for the entire daytime period of observation. Metabolic rate was determined from ambient temperature and the regression lines from respirometry trials (Fig. 1) accounting for periods of torpor and euthermia. The lines correspond to the expected field metabolic rate if the bats had remained strictly euthermic (dashed line), or torpid (solid line) based on respirometry trials. The secondary y-axis converts metabolic rate to the mass of fat required.

The cumulative amount of time spent in torpor was related to T_a_. On cooler days, bats spent more time in torpor (*F*_1,16_ = 16.09, *P* = 0.001; Fig. 3a), and thus saved more energy (*F*_1,16_ = 14.09, *P* = 0.002; Fig. 3b). At lower T_a_, the potential energy savings is greater due to the increasing difference between TMR and RMR (Fig. 1). Consequently, variation in energy savings results from the combined (but not independent) effects of lower T_a_ and increased time in torpor. When accounting for torpor use, bats saved 8–91% of the energy requirement compared to maintaining euthermic body temperature (Fig. 3b). Bats spent more time in torpor on cooler days (Fig. 3a), but the total daytime energy expenditure was independent of T_a_ (*F*_1,16_ = 0.58, *P* = 0.46; Fig. 4).

## Discussion

Migrating bats used torpor to save up to 91% of the energy they would have otherwise expended to defend euthermic body temperature. Unlike homeothermic birds, where ambient temperature greatly affects thermoregulatory costs [4], bats did not expend more energy on colder days. Furthermore, bats could use torpor facultatively to mitigate the energetic consequences of day-to-day variation in ambient temperature conditions. With a flexible strategy of heterothermy, bats are not subject to greater energy demands (and hence reduced net refuelling rate) on cooler days, and therefore are able to maintain short stopover durations. Thus, torpor-assisted migration enables migrating bats to save both time and energy.

Traditional interpretations of torpor have focussed on using torpor in cases of energetic emergency, but there are many recent examples of adaptive reasons to use torpor [23], [24]. We did not observe an effect of body composition on the use of torpor in the field. However, it is possible that above or below certain thresholds of body composition bats may respond by decreasing or increasing their use of torpor. The thermoregulatory patterns we observed are consistent with bats facultatively using torpor to save energy in anticipation of future demand. This reduction in energy expenditure affects overall energy balance in a similar way to the increased energy intake of migratory birds facing greater future migration costs [25].

In considerations of migratory stopover using optimal migration theory [26], there is thought to be trade-offs between time, energy, and safety. Migrants are described as being either time, energy, or predation risk minimizers. We predict that torpor-assisted migration will modulate these trade-offs. The efficiency and success of bird migration is largely dependent on conditions experienced at stopover [27]. Weather can impact foraging conditions (energy input) and thermoregulatory costs (energy output). Net energy balance strongly affects stopover duration, and therefore the pace of migration, physiological condition, and perhaps even survival and reproductive success [27]. By lessening energy expenditure during inactive periods through the use of torpor, bats may reduce their need to refuel during migration (note the similarities with bats increasing torpor use prior to hibernation [28]). With lower energy expenditure and shorter refuelling periods, migration may be completed more quickly. Based on allometrically predicted flight cost (1.5 W, assuming 10.5 g body mass; [21]), and estimated flight speed (9.04 m s^−1^; [10]), the energy saved (3351–14895 J; this study) by using torpor on cooler days (mean T_a_<20°C) would provide enough energy to fly an additional 20–90 km per night. Computer simulation predictions [10] indicate this additional distance may represent up to 36% of the distance travelled each night. If torpor increases the speed of migration (less time spent in unfamiliar territory) or if it reduces predation exposure (less active time exposed to predators), migrating bats may have a higher rate of survival compared to migrating birds. Recent mark-recapture survival estimates for a migratory bat (*Nyctalus leisleri*; [29]) suggest that migration-related mortality is much lower than similar estimates for a migrating songbird (*Setophaga caerulescens*; [30]).

Torpor-assisted migration provides clear energy and time benefits (and possibly survival benefits) for migrating bats, particularly given time constraints faced by these nocturnal animals. Every bat we tracked used torpor to some degree, yet a number of factors may affect the use of torpor in migrating bats. Although we were not able to directly observe free-living bats in their daytime roosts, migrating silver-haired bats have been documented to use a wide variety of roosts at this site [10]. Bats roosting in more or less exposed locations would experience a different range of T_a_. Furthermore, roost selection will affect the ability of bats to rewarm passively [31]. In the morning, increasing T_a_ would allow animals to rewarm passively with T_a_ and solar radiation [32]. Passive rewarming may reduce arousal cost by>50% (e.g., [31], [33], [34]). Without knowledge of roost microclimate, we are not able to account for passive rewarming. However, if mechanisms of enhanced passive rewarming were important, actual energy savings realized by bats will be greater than we have conservatively reported. Therefore roost microclimate and passive rewarming should be important components for future studies.

The energy saved through torpor use would allow bats to undergo minimal refuelling *en route* and potentially to rely heavily on nutrient stores deposited prior to migration. This could be particularly relevant for species where fat deposited prior to migration is used for subsequent hibernation (e.g., [35]). If this is the case, there is a potential for strong carryover effects from post-breeding conditions to subsequent periods of the annual cycle. An interesting possibility to consider is whether bats could further reduce their need to forage at stopover sites by foraging on the wing during migratory flight. Insectivorous bats can fuel flight with nutrients ingested during current foraging bouts [36], but we suggest it is unlikely they would use this strategy during migratory flights. Two key factors in the definition of migration as described by [37] are undistracted and direct movements. Erratic flight in search of insect prey violates both these criteria. We suggest that bats are more likely to engage in foraging either prior to or following flight each night of travel, allowing for undistracted and direct migratory flight through the night. Evidence from migrating *Pipistrellus nathusii* supports this hypothesis. Bats captured early in the night exhaled stable carbon isotopes indicative of supporting metabolism with insect prey, but carbon isotope signatures of bats captured later at night indicated the bats relied on more on endogenous adipose stores [38]. These results suggest the bats may have foraged in the evening prior to a migratory flight. Further research is required to determine when, where, and how bats deposit adipose stores to fuel their migration.

Perhaps the most important factor to consider when discussing the cost-benefit tradeoffs of a torpor-assisted migration strategy is the reproductive condition of female bats. During spring migration, females migrate north while pregnant. The coincidence of migration and pregnancy affects both time and energy budgets. In addition to the energetic costs of pregnancy, females also face time pressures to reach summer grounds to give their pups a longer growing season. As we have demonstrated, using torpor during inactive periods reduces time and energy for both sexes during autumn migration. However the costs of reduced body temperature for developing foetuses may negate any benefits to mothers. Accordingly, female hoary bats captured during spring migration rarely used torpor when cold challenged, while males readily lowered body temperature with decreasing T_a_ [8]. The costs of heterothermy for unborn pups may be dependent on the developmental stage. Torpor has been observed in female silver-haired bats (most likely pregnant) as they approach the northward end of spring migration [7] and in pregnant female hoary bats upon reaching the summer grounds [39]. The full implications and interactions of torpor-assisted migration and pregnancy remain to be elucidated.

Bats are well known for their heterothermic abilities, a strategy that is uncommon in birds [40]. However, torpor-assisted migration is a strategy that may not be restricted to bats, and may in fact be one of the important ‘other functions of torpor’ [24]. Hummingbirds are known to use torpor during migration which may lead to similar energetic benefits [41]. Other migratory bird lineages, including caprimulgids and swifts, are known to use torpor at other times of year [40], and may follow a similar migration strategy. Furthermore, torpor-assisted migration may be a specialized case of a more general pattern of heterothermic migration strategies. In birds not known to use torpor, shallow hypothermia has been suggested as an energy-saving strategy [40], [42], [43]. Although thermoregulatory strategies have been largely overlooked in studies of migration physiology, our research indicates that future studies should consider the role of heterothermy in the energetics of both bat and bird migration.

## Supporting Information

S1 Table **Respirometry data.** Data from respirometry measurements of silver-haired bats *Lasionycteris noctivagans* captured during autumn migration.
(pdf)

S2 Table **Telemetry and energetic calculations data.** Radiotracking and energetic calculations data for silver-haired bats *Lasionycteris noctivagans* captured during autumn migration.
(pdf)
