# Supplementary material for: Bats on a Budget: Torpor-Assisted Migration Saves Time and Energy
Source: PLoS One. 2014 Dec 31;9(12):e115724. doi: 10.1371/journal.pone.0115724 (PMC4281203; doi:10.1371/journal.pone.0115724)
Supplement: S1 Table — Respirometry data. Data from respirometry measurements of silver-haired bats Lasionycteris noctivagans captured during autumn migration. (pdf) [file pone.0115724.s001.pdf]

**Table S1. Respirometry Data.** Data from respirometry measurements of silver-haired bats *Lasionycteris noctivagans* captured during autumn migration. Each bat was measured at two different temperatures (see Materials and Methods). Fat mass and lean mass were measured by quantitative magnetic resonance prior to respirometry.

| Bat ID | Respirometry Temperature (°C) | RMR (mL $O_2$ $hr^{-1}$ $g^{-1}$) | TMR (mL $O_2$ $hr^{-1}$ $g^{-1}$) | Age | Sex | Body Mass (g) | Forearm Length (mm) | Fat Mass (g) | Lean Mass (g) |
|---|---|---|---|---|---|---|---|---|---|
| LANO-17 | 20.0 | | 0.33 | sub-adult | female | 10.7 | 42.00 | 1.21 | 8.38 |
| LANO-17 | 22.5 | 2.18 | 0.29 | sub-adult | female | 10.7 | 42.00 | 1.21 | 8.38 |
| LANO-29 | 25.0 | 4.10 | | sub-adult | female | 11.5 | 42.25 | 1.45 | 8.64 |
| LANO-29 | 22.5 | 2.59 | | sub-adult | female | 11.5 | 42.25 | 1.45 | 8.64 |
| LANO-30 | 25.0 | 1.99 | | sub-adult | female | 11.3 | 42.70 | 1.30 | 8.88 |
| LANO-30 | 22.5 | 2.53 | | sub-adult | female | 11.3 | 42.70 | 1.30 | 8.88 |
| LANO-31 | 25.0 | 2.20 | | sub-adult | female | 10.2 | 41.30 | 0.92 | 8.38 |
| LANO-31 | 22.5 | 2.81 | | sub-adult | female | 10.2 | 41.30 | 0.92 | 8.38 |
| LANO-37 | 20.0 | 3.77 | | sub-adult | female | 11.1 | 40.60 | 1.59 | 8.41 |
| LANO-37 | 22.5 | 3.76 | 0.93 | sub-adult | female | 11.1 | 40.60 | 1.59 | 8.41 |
| LANO-38 | 20.0 | 4.12 | | sub-adult | male | 10.9 | 41.75 | 1.37 | 8.45 |
| LANO-38 | 22.5 | 2.67 | | sub-adult | male | 10.9 | 41.75 | 1.37 | 8.45 |
| LANO-40 | 20.0 | 3.34 | | sub-adult | male | 11.3 | 40.60 | 1.76 | 8.41 |
| LANO-40 | 22.5 | 3.22 | | sub-adult | male | 11.3 | 40.60 | 1.76 | 8.41 |
| LANO-41 | 20.0 | 3.08 | | adult | male | 10.6 | 39.55 | 1.32 | 8.23 |
| LANO-41 | 22.5 | 1.95 | | adult | male | 10.6 | 39.55 | 1.32 | 8.23 |
| LANO-42 | 15.0 | 4.66 | | sub-adult | female | 11.4 | 41.30 | 1.20 | 8.22 |
| LANO-42 | 17.5 | 3.53 | | sub-adult | female | 11.4 | 41.30 | 1.20 | 8.22 |
| LANO-43 | 15.0 | 5.41 | | sub-adult | male | 10.3 | 41.00 | 1.24 | 7.81 |
| LANO-43 | 17.5 | 4.38 | 0.25 | sub-adult | male | 10.3 | 41.00 | 1.24 | 7.81 |
| LANO-44 | 15.0 | 3.19 | 0.34 | sub-adult | male | 8.9 | 39.80 | 0.87 | 6.87 |
| LANO-44 | 17.5 | 7.51 | 0.13 | sub-adult | male | 8.9 | 39.80 | 0.87 | 6.87 |
| LANO-51 | 15.0 | 5.32 | 0.05 | adult | male | 10.9 | 41.00 | 0.59 | 8.76 |
| LANO-51 | 17.5 | 4.33 | 0.15 | adult | male | 10.9 | 41.00 | 0.59 | 8.76 |
| LANO-54 | 25.0 | 1.91 | | sub-adult | female | 10.2 | 39.80 | 1.15 | 7.71 |
| LANO-54 | 27.5 | 1.39 | | sub-adult | female | 10.2 | 39.80 | 1.15 | 7.71 |
| LANO-58 | 27.5 | 1.92 | | sub-adult | female | 11.3 | 39.95 | 0.88 | 9.13 |
| LANO-58 | 25.0 | 2.83 | | sub-adult | female | 11.3 | 39.95 | 0.88 | 9.13 |
| LANO-59 | 27.5 | 1.41 | | adult | male | 9.5 | 39.10 | 1.03 | 7.36 |
| LANO-59 | 25.0 | 1.57 | | adult | male | 9.5 | 39.10 | 1.03 | 7.36 |
| LANO-63 | 17.5 | 3.23 | | adult | female | 12.1 | 42.70 | 1.69 | 9.02 |
| LANO-63 | 15.0 | 6.75 | 0.07 | adult | female | 12.1 | 42.70 | 1.69 | 9.02 |
| LANO-64 | 17.5 | 3.60 | 0.46 | sub-adult | female | 9.9 | 42.05 | 0.43 | 8.30 |
| LANO-64 | 15.0 | 9.38 | 0.20 | sub-adult | female | 9.9 | 42.05 | 0.43 | 8.30 |
| LANO-65 | 17.5 | 3.85 | 0.44 | adult | female | 11.5 | 42.45 | 0.72 | 9.25 |
| LANO-65 | 15.0 | 3.31 | 0.20 | adult | female | 11.5 | 42.45 | 0.72 | 9.25 |
| LANO-67 | 17.5 | 5.84 | 0.37 | sub-adult | female | 9.9 | 40.45 | 0.68 | 7.97 |
| LANO-67 | 15.0 | 6.36 | 0.12 | sub-adult | female | 9.9 | 40.45 | 0.68 | 7.97 |
| LANO-71 | 25.0 | 1.38 | | sub-adult | female | 10.3 | 42.90 | 0.77 | 8.25 |
| LANO-71 | 27.5 | 1.10 | | sub-adult | female | 10.3 | 42.90 | 0.77 | 8.25 |
| LANO-8 | 20.0 | 3.59 | | sub-adult | male | 9 | 39.65 | 0.61 | 7.38 |
| LANO-8 | 22.5 | 1.78 | | sub-adult | male | 9 | 39.65 | 0.61 | 7.38 |
| LANO-9 | 20.0 | 3.42 | | sub-adult | male | 10.1 | 43.40 | 0.56 | 8.34 |
| LANO-9 | 22.5 | 2.37 | | sub-adult | male | 10.1 | 43.40 | 0.56 | 8.34 |
