# Supplementary material for: Bats on a Budget: Torpor-Assisted Migration Saves Time and Energy
Source: PLoS One. 2014 Dec 31;9(12):e115724. doi: 10.1371/journal.pone.0115724 (PMC4281203; doi:10.1371/journal.pone.0115724)
Supplement: S2 Table — Telemetry and energetic calculations data. Radiotracking and energetic calculations data for silver-haired bats Lasionycteris noctivagans captured during autumn migration. (pdf) [file pone.0115724.s002.pdf]

Table S2. Telemetry and energetic calculations data. Radiotracking and energetic calculations data for silver-haired bats *Lasionycteris noctivagans* captured during autumn migration. See main text for details. Note that data are included from two bats each measured on two separate days (LANO-55, LANO-74).

Bat ID	Date	Sex	Age	Body mass (g)	Forearm length (mm)	Fat mass (g)	Lean mass (g)	Time euthermic (h)	Time not euthermic (h)	Mean T_a (°C)	Metabolic rate including heterothermy (W g^{-1})	Metabolic rate assuming homeothermy (W g^{-1})	Energy Savings (%)
LANO-1	Aug 26	female	sub-adult	10.0	41.65	0.79	8.06	6.29	4.34	21.8	0.0116	0.0170	31.7
LANO-12	Aug 28	male	sub-adult	9.8	40.10	0.95	7.64	4.26	5.62	22.0	0.0097	0.0166	41.6
LANO-13	Aug 28	male	sub-adult	9.5	41.20	0.52	7.78	4.24	6.30	21.9	0.0086	0.0168	49.1
LANO-15	Aug 28	female	sub-adult	10.8	40.55	1.07	8.56	3.18	7.13	21.9	0.0073	0.0167	56.3
LANO-16	Aug 28	female	sub-adult	10.0	39.90	0.68	8.21	6.25	3.45	22.1	0.0117	0.0165	28.9
LANO-27	Aug 29	male	sub-adult	11.5	40.25	1.12	9.17	6.67	1.51	22.0	0.0146	0.0166	12.3
LANO-32	Sep 01	male	adult	10.3	41.15	0.73	8.36	10.84	2.61	23.9	0.0110	0.0133	17.3
LANO-34	Sep 03	female	sub-adult	10.6	41.60	0.86	8.39	10.58	1.95	27.4	0.0067	0.0085	21.0
LANO-50	Sep 06	male	sub-adult	10.3	40.60	1.08	8.06	0.26	10.04	15.6	0.0025	0.0283	91.3
LANO-55 a	Sep 07	male	adult	11.4	41.70			2.77	9.31	16.9	0.0085	0.0258	67.0
LANO-55 b	Sep 08	male	adult	11.4	41.70			0.04	12.16	18.3	0.0022	0.0232	90.7
LANO-56	Sep 07	male	sub-adult	9.7	40.00			0.35	11.29	16.9	0.0040	0.0258	84.7
LANO-69	Sep 11	female	adult	11.0	40.80	1.35	8.36	10.54	1.47	20.6	0.0176	0.0191	8.0
LANO-70	Sep 12	male	sub-adult	11.0	40.70	1.10	8.65	5.66	5.96	21.4	0.0102	0.0178	42.6
LANO-72	Sep 13	male	sub-adult	10.3	42.75	0.87	8.21	7.88	4.86	22.5	0.0117	0.0158	25.9
LANO-73	Sep 14	female	sub-adult	11.5	43.00	1.03	9.18	6.30	5.36	16.8	0.0142	0.0260	45.5
LANO-74 a	Sep 14	female	sub-adult	11.5	41.60	1.67	8.74	8.34	4.21	16.4	0.0203	0.0268	24.1
LANO-74 b	Sep 16	female	sub-adult	11.5	41.60	1.67	8.74	0.70	11.71	12.8	0.0042	0.0332	87.4
